# Simultaneous determination of itraconazole and its CYP3A4-mediated metabolites including *N*-desalkyl itraconazole in human plasma using liquid chromatography-tandem mass spectrometry and its clinical application

**DOI:** 10.1186/s40780-020-00167-7

**Published:** 2020-05-04

**Authors:** Yumi Imoto, Yasuaki Mino, Takafumi Naito, Takaaki Ono, Junichi Kawakami

**Affiliations:** 1 Department of Hospital Pharmacy, Hamamatsu University School of Medicine, 1-20-1 Handayama, Higashi-ku, Hamamatsu, 431-3192 Japan; 2 Division of Hematology, Internal Medicine 3, Hamamatsu University School of Medicine, 1-20-1 Handayama, Higashi-ku, Hamamatsu, 431-3192 Japan

**Keywords:** Itraconazole, Metabolites, LC-MS/MS, Human plasma, Pharmacokinetics

## Abstract

**Background:**

Itraconazole (ITZ), a triazole antifungal agent, is metabolized to hydroxy-ITZ (OH-ITZ), keto-ITZ (KT-ITZ), and *N*-desalkyl ITZ (ND-ITZ) by cytochrome P450 3A4. The pharmacokinetics of ND-ITZ remain largely unknown due to the lack of an accurate and reliable determination method. This study aimed to develop a simultaneous determination method for ITZ and its three major metabolites including ND-ITZ in human plasma using isocratic liquid chromatography coupled to tandem mass spectrometry and then apply the method in a clinical setting.

**Methods:**

Plasma specimens were pretreated by protein precipitation with acetonitrile. The supernatant was separated on a 3-μm particle octadecyl silane column (75 × 2.0 mm I.D.) in an isocratic elution of acetonitrile and 5 mM ammonium acetate (pH 6.0) (57:43, v/v). The method was applied to 10 patients treated with oral ITZ.

**Results:**

The calibration curves of ITZ, OH-ITZ, KT-ITZ, and ND-ITZ were linear over the concentration ranges of 15–1500, 15–1500, 1–100, and 1–100 ng/mL, respectively. The pretreatment recoveries and matrix factors were 90.1–102.2% and 99.1–102.7%. Their intra- and inter-assay accuracies and imprecisions were 94.1–106.7% and 0.3–4.4%. The plasma concentrations of ITZ, OH-ITZ, KT-ITZ, and ND-ITZ 12 h after dosing ranged from 32.5–1127.1, 19.0–1166.7, 1.1–5.4, and 3.5–28.3 ng/mL, respectively, in immunocompromised patients.

**Conclusions:**

This study developed a simultaneous determination method for concentrations of ITZ and its three metabolites including ND-ITZ in a clinical setting.

## Background

Itraconazole (ITZ), a triazole antifungal agent, is commonly used for the prevention and treatment of fungal infections [1, 2]. The plasma concentration of ITZ showed a large variation between patients treated with its capsule or oral solution formulation in a recent report [3]. The clinical significance of interindividual differences in the plasma concentration of ITZ is still controversial. An optimal ITZ concentration for prevention from fungal infection and for treatment was proposed [4]. The required concentration of ITZ and hydroxy-ITZ (OH-ITZ) as a total was also advocated for the prevention of fungal infections [5]. Therapeutic drug monitoring can be considered because the ITZ concentration differs between individuals, probably owing to metabolism variability [5–7].

**Fig. 1:**
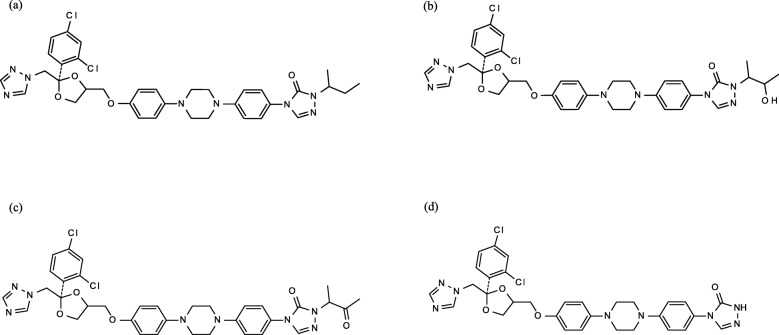
The chemical structures of (**a**) itraconazole, (**b**) hydroxy itraconazole, (**c**) keto itraconazole, and (**d**) *N*-desalkyl itraconazole

ITZ is mainly metabolized to OH-ITZ by liver cytochrome P450 (CYP) 3A4 [5, 8]. OH-ITZ is further converted to keto-ITZ (KT-ITZ) and to *N*-desalkyl ITZ (ND-ITZ) via CYP3A4 metabolism (Fig. 1) [8–10]. Only ITZ and OH-ITZ were reported to have antifungal activity [11, 12]. KT-ITZ, in addition to ITZ and OH-ITZ, inhibits CYP3A4 activity. ND-ITZ exhibited unbound IC_50_ values of 0.4 nM, when coincubated with human liver microsomes and midazolam [8], however, it is unclear whether ND-ITZ inhibits CYP3A4 or does not in clinical practice. ITZ inhibits P-glycoprotein, while OH-ITZ and KT-ITZ inhibit P-glycoprotein, organic anion transporting polypeptide 1B1 and 1B3, and multidrug and toxin extrusion protein 1 [13]. To date, the pharmacokinetics of ND-ITZ have not been fully characterized, most likely because of the lack of a determination method. Pharmacokinetic data may provide valuable information regarding ITZ treatment and potential drug-drug interactions.

A simultaneous determination method of ITZ and OH-ITZ in human plasma using a conventional high-performance liquid chromatography-ultra-violet detection system was reported [14]. The plasma concentrations of KT-ITZ and ND-ITZ were so low that liquid chromatography-mass spectrometry (LC-MS) or tandem mass spectrometry (MS/MS) analysis was essential for their determination. More selective and sensitive determination methods of ITZ and OH-ITZ that employed MS detection have been reported [2, 14–19]. However, their methods were not intended for use in clinical settings due to various technical limitations. Thus far, only one study is available on a simultaneous determination method for ITZ, OH-ITZ, KT-ITZ, and ND-ITZ in healthy subjects [15]. This previous study employed a solid-supported liquid extraction and high pressure LC system. The method was applied only in healthy subjects and not in patients. A more convenient method is needed for clinical settings.

This study aimed to develop a simultaneous determination method for ITZ and its three major metabolites including ND-ITZ using an isocratic LC-MS/MS method in human plasma obtained from immunocompromised patients.

## Methods

### Materials

ITZ, OH-ITZ, KT-ITZ, ND-ITZ, and ITZ*-d9* as an internal standard (IS) were obtained from Toronto Research Chemicals Inc. (Toronto, Ontario, Canada). HPLC-grade acetonitrile and methanol were purchased from Wako Pure Chemicals (Osaka, Japan). Stock solutions of ITZ (50 μg/mL), OH-ITZ (50 μg/mL), KT-ITZ (50 μg/mL), ND-ITZ (2 μg/mL), and ITZ*-d9* (50 μg/mL) were prepared with methanol. Stock solutions of ITZ, OH-ITZ, KT-ITZ, and ND-ITZ were diluted in acetonitrile to yield their standard solutions.

### Sample pretreatment

Plasma treated with ethylenediaminetetraacetic acid was obtained by the centrifugation of blood at 1670×*g* at 4 °C for 10 min and then pooled at − 80 °C until sample pretreatment. Four hundred microliters of acetonitrile and 100 μL of IS solution (500 ng/mL) were added to 100 μL aliquots of plasma. After mixing and ultrasonication, the samples were centrifuged at 18,000×*g* at 4 °C for 20 min, and then 450 μL of the supernatant was dried using rotary vacuum evaporation. The residues were dissolved with 150 μL of mobile phase. After mixing and ultrasonication, the mixtures were centrifuged at 18,000×*g* for 20 min at 4 °C. The supernatants were filtered with a Millex-LH syringe filter (0.45 μm, 4 mm, Merck Millipore Ltd., Billerica, MA, USA) before injection into the LC.

### Chromatographic conditions

ITZ, OH-ITZ, KT-ITZ, ND-ITZ, and IS in human plasma were separated using an LC system (NexeraX2, Shimadzu Corporation, Kyoto, Japan). The LC system consisted of a CBM-20A, DGU-20A_5R_, LC-30AD_XR_ NexeraX2, SIL-30 AC NexeraX2, CTO-20 AC, and FCV 20AH_2_. A 3-μm particle octadecyl silane (ODS) column (TSKgel ODS-100 V, 75 × 2.0 mm I.D., Tosoh, Tokyo, Japan) was used to separate ITZ and its metabolites. The mobile phase composed of acetonitrile and 5 mM ammonium acetate (pH 6.0) (57:43, v/v). The flow rate was 0.2 mL/min. The autoinjector and the column temperature were set at 4 °C and 40 °C, respectively. The injection volume of the samples was 2 μL.

### Mass spectrometric conditions

The column effluent was monitored using a triple quadrupole mass spectrometer (LCMS-8050, Shimadzu Corporation) equipped with an electrospray probe in positive ionization mode. It was controlled with LabSolutions ver 5.85 software (Shimadzu Corporation). In this study, multiple reaction monitoring (MRM) using a triple-quadrupole mass spectrometer was used. MRM enables the setting of multiple channels in one measurement. LC-MS/MS (MRM) is more suitable than LC-MS for analysis involving more contaminants. Analyses conducted using LC-MS/MS have higher sensitivity than LC-MS. The MRM was checked using a dwell time of 200 milliseconds for each compound: ITZ, 706.05/393.05; OH-ITZ, 721.15/408.15; KT-ITZ, 719.10/406.10; ND-ITZ, 649.10/376.15; and ITZ*-d9*, 714.25/401.15. Samples were injected to the interface through a turbo ion spray with the temperature set at 350 °C. The collision-induced-dissociation gas, drying gas, nebulizer gas, and heating gas were set at 17 kPa, 10 L/min, 3 L/min, and 10 L/min, respectively. Collision energies for ITZ, OH-ITZ, KT-ITZ, ND-ITZ, and ITZ*-d9* were − 38, − 37, − 37, − 31, and − 36 V, respectively.

### Method validation

Assay selectivity was evaluated by analyzing six independent drug-free plasma samples. Calibration curves were acquired by plotting the measured peak area ratios of ITZ, OH-ITZ, KT-ITZ, and ND-ITZ to IS. The linearities of ITZ, OH-ITZ, KT-ITZ, and ND-ITZ were 15–1500, 15–1000, 1–100, and 1–100 ng/mL, respectively. Calibration standards were used with drug-free pooled plasma (Kohjin-Bio Co., Ltd., Saitama, Japan). The final concentrations of ITZ and OH-ITZ in plasma were 15, 45, 60, 150, 300, 600, 900, 1200, and 1500 ng/mL, while those of KT-ITZ and ND-ITZ were 1, 3, 4, 10, 20, 40, 60, 80, and 100 ng/mL, respectively. Quality control (QC) samples at low, medium, and high concentrations containing ITZ and OH-ITZ (45, 300, 1200 ng/mL), KT-ITZ and ND-ITZ (3, 20, and 80 ng/mL) were prepared. The lower limits of quantification (LLOQ) were defined as analyte concentrations at which the relative standard deviation does not exceed 20%. Pretreatment recovery and matrix effect were assessed by three replicates of spiked human plasma at the concentration of the QC samples. The accuracies of ITZ, OH-ITZ, KT-ITZ, and ND-ITZ in human plasma were determined by assessing the analytical recovery of known amounts of plasma specimens. The accuracies and imprecisions were calculated for three QC samples in plasma. The intra- and inter-assay imprecisions were expressed as the relative standard deviation. The integrity of the dilution was monitored by diluting high concentration samples (ITZ and OH-ITZ, 6000 ng/mL and KT-ITZ and ND-ITZ, 400 ng/mL) 5 times with drug-free human plasma in order to demonstrate the accuracy and precision of these diluted samples compared to QC samples (ITZ and OH-ITZ, 1200 ng/mL and KT-ITZ and ND-ITZ, 80 ng/mL). The carryover was assessed by measuring detector signals of blank plasma after the higher QC samples. Detector signals less than 20% of LLOQ signals were regarded as the acceptable limits. The stabilities of analyte in plasma were calculated by comparing peak areas after 24 h of storage at 4 °C and room temperature with initial peak area. Long-term stabilities in plasma at − 80 °C were evaluated after 1 month. Analytical stabilities in injection solutions were calculated by comparing peak areas after 12 h of storage at 4 °C with initial peak area.

### Patients and pharmacokinetic evaluation

Ten Japanese immunocompromised patients with hematological disorder at Hamamatsu University Hospital (Hamamatsu, Japan) were enrolled. The patients received 200 mg of oral ITZ capsule formulation (Itrizole® capsule, Janssen Pharmaceutical K.K., Tokyo) once daily at bedtime for the prevention of fungal infections. The exclusion criteria for the study were as follows: patients (1) co-treated with another triazole antifungal drug; (2) whose serum total bilirubin was more than 2.0 mg/dl before starting ITZ treatment; (3) whose serum creatinine was more than 1.5 mg/dl before starting ITZ treatment; and (4) with poor adherence based on interviews and medical records. Two-mL blood samples were collected 12 h after the dose on the 14th day or later after initiation of therapy.

## Results

### Separation and selectivity

**Fig. 2:**
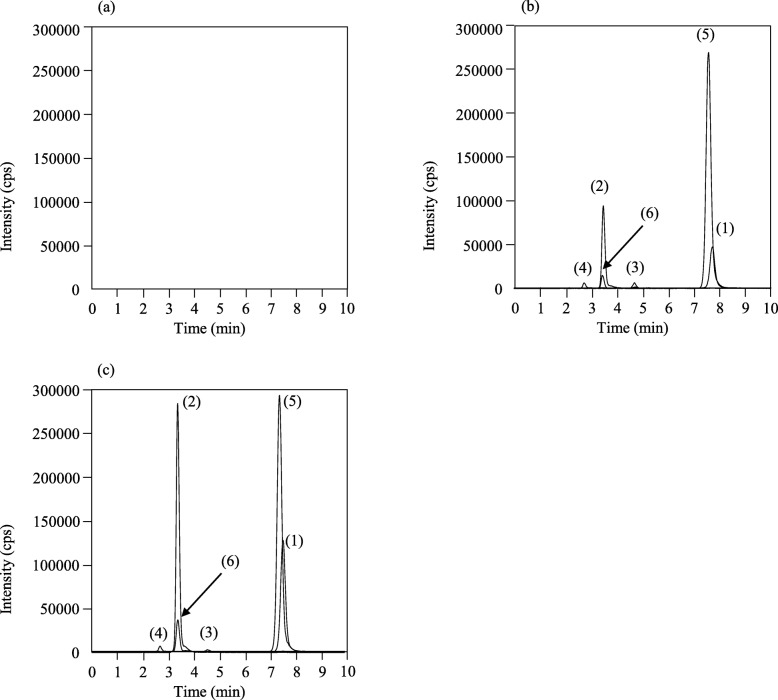
The LC-MS/MS chromatograms of a drug-free plasma sample (**a**), plasma sample spiked with 300 ng/mL itraconazole and 300 ng/mL hydroxy itraconazole, 20 ng/mL keto itraconazole and 20 ng/mL *N*-desalkyl itraconazole (**b**), and a plasma sample from a patient receiving 200 mg of oral itraconazole once daily at bedtime for prevention of fungal infections, whose concentrations were as follows: itraconazole, 729 ng/mL; hydroxy itraconazole, 859 ng/mL; keto itraconazole, 5.4 ng/mL and *N*-desalkyl itraconazole, 16.6 ng/mL (**c**). Peaks of itraconazole, hydroxy itraconazole, keto itraconazole, *N*-desalkyl itraconazole, and itraconazole*-d9* as internal standard, are (1), (2), (3), (4), and (5), respectively. Peaks of isotopes are observed for hydroxy itraconazole and keto itraconazole (6). The MRM transitions are as follows: itraconazole, 706.05/393.05; hydroxy itraconazole, 721.15/408.15; keto itraconazole, 719.10/406.10; *N*-desalkyl itraconazole, 649.10/376.15; and internal standard, 714.25/401.15

ITZ, OH-ITZ, KT-ITZ, ND-ITZ, and ITZ*-d9* were eluted at 7.6, 3.6, 4.7, 2.8, and 7.3 min, respectively. Total run time was 10 min. No interfering peaks were observed in six independent drug-free plasma specimens (Fig. 2).

### Calibration curve and sensitivity

**Fig. 3:**
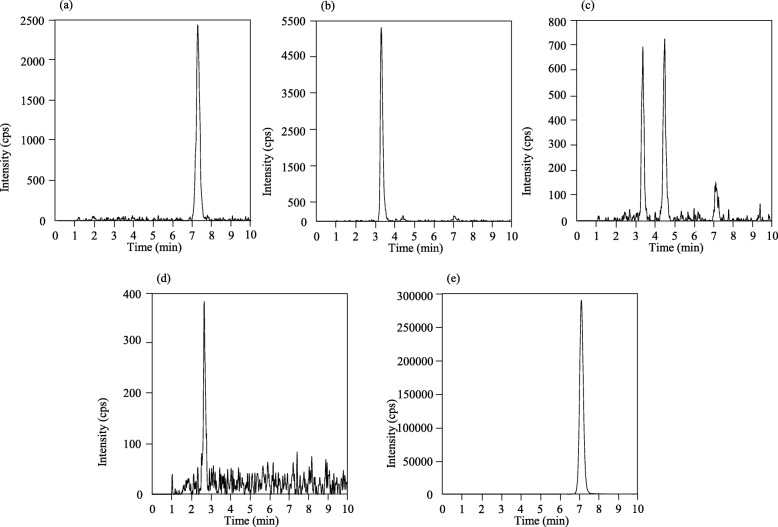
Representative LLOQ chromatograms of itraconazole (**a**), hydroxy itraconazole (**b**), keto itraconazole (**c**), *N*-desalkyl itraconazole (**d**), and itraconazole-d9 as an internal standard. (**e**)

The calibration curves of ITZ, OH-ITZ, KT-ITZ, and ND-ITZ in human plasma were linear over the concentration ranges of 15–1500, 15–1500, 1–100, and 1–100 ng/mL, respectively. Their coefficients of correlation were greater than 0.999 (Table 1). The LLOQs of ITZ, OH-ITZ, KT-ITZ, and ND-ITZ in human plasma were 15, 15, 1, and 1 ng/mL, respectively (*n* = 6) (Fig. 3). The intra- and inter-assay accuracies at LLOQ of ITZ, OH-ITZ, KT-ITZ, and ND-ITZ were 99.8 and 94.1%, 97.6 and 98.3%, 100.9 and 102.7%, and 106.7 and 103.5%, respectively. The intra- and inter-assay imprecisions at LLOQ of ITZ, OH-ITZ, KT-ITZ, and ND-ITZ were 0.8 and 4.0%, 3.3 and 6.5%, 5.4 and 10.3%, and 4.0 and 8.2%, respectively (Table 2).

**Table 1:** Calibration curve ranges of ITZ and its metabolites and their individual correlation coefficients

	Calibration curve range (ng/mL)	LLOQ (ng/mL)	Correlation coefficients
ITZ	15–1500	15	0.999
OH-ITZ	15–1500	15	1.000
KT-ITZ	1–100	1	0.999
ND-ITZ	1–100	1	0.999

**Table 2:** LC-MS/MS assay analytical parameters of ITZ and its metabolites in human plasma

	Theoretical value (ng/mL)	Intra-assay			Inter-assay		
		mean ± SD (ng/mL)	Accuracy (%)	RSD (%)	mean ± SD (ng/mL)	Accuracy (%)	RSD (%)
ITZ	LLOQ (15)	15.0 ± 0.1	99.8	0.8	14.1 ± 0.6	94.1	4.0
	L (45)	45.4 ± 0.8	100.8	1.8	45.4 ± 1.1	100.9	2.4
	M (300)	297 ± 2.5	99.1	0.8	305 ± 4.6	101.8	1.5
	H (1200)	1201 ± 16	100.1	1.4	1213 ± 26	101.1	2.1
OH-ITZ	LLOQ (15)	14.6 ± 0.5	97.6	3.3	14.7 ± 1.0	98.3	6.5
	L (45)	45.0 ± 1.3	100.1	3.0	45.8 ± 0.6	101.7	1.3
	M (300)	299 ± 1.8	99.8	0.6	303 ± 7.4	101.1	2.4
	H (1200)	1197 ± 21	99.7	1.8	1214 ± 34	101.1	2.8
KT-ITZ	LLOQ (1)	1.01 ± 0.05	100.9	5.4	1.03 ± 0.11	102.7	10.3
	L (3)	3.00 ± 0.05	100.0	1.8	3.02 ± 0.11	100.6	3.7
	M (20)	19.9 ± 0.4	99.5	2.0	19.8 ± 1.0	99.1	4.9
	H (80)	80.2 ± 4.0	100.2	4.9	81.1 ± 2.1	101.4	2.7
ND-ITZ	LLOQ (1)	1.07 ± 0.04	106.7	4.0	1.04 ± 0.08	103.5	8.2
	L (3)	2.99 ± 0.11	99.6	3.8	2.97 ± 0.06	98.8	2.2
	M (20)	20.2 ± 1.6	99.9	8.1	20.5 ± 0.8	102.6	3.8
	H (80)	80.0 ± 3.2	100.0	3.9	82.2 ± 4.4	102.7	5.3

*ITZ* itraconazole, *OH-ITZ* hydroxy-ITZ, *KT-ITZ* keto-ITZ, *ND-ITZ N*-desalkyl ITZ, *LLOQ* lower limit of quantification, *L* low, *M* medium, *H* high, *SD* standard deviation; and *RSD* relative standard deviation

### Pretreatment recovery, matrix effect and carryover

The mean pretreatment recoveries of ITZ, OH-ITZ, KT-ITZ, and ND-ITZ were, 98.4–101.8%, 96.9–102.2%, 90.1–98.4%, and 93.0–98.5%, respectively. The analytes did not exhibit any matrix effects in human plasma (mean, 100.6–101.8% for ITZ, 99.1–100.5% for OH-ITZ, 99.7–100.2% for KT-ITZ, and 99.1–102.7% for ND-ITZ). The obtained carryover effects were within acceptable limits.

### Assay accuracy and imprecision in human plasma

The intra- and inter-assay accuracies and imprecisions in human plasma were shown in Table 2. The intra- and inter-assay accuracies of ITZ, OH-ITZ, KT-ITZ, and ND-ITZ were 99.1–100.8% and 100.9–101.8%, 99.7–100.1% and 101.1–101.7%, 99.5–100.2% and 99.1–101.4%, and 99.6–100.0% and 98.8–102.7%, respectively. The intra- and inter-assay imprecisions of ITZ, OH-ITZ, KT-ITZ, and ND-ITZ were 0.8–1.8% and 1.5–2.4%, 0.6–3.0% and 1.3–2.8%, 1.8–4.9% and 2.7–4.9%, and 3.8–8.1% and 2.2–5.3%, respectively. The accuracy and precision of diluted samples in plasma mimicked the expected dilutions (% of QC samples, ITZ, 101.3 ± 1.6%; OH-ITZ, 100.3 ± 1.2%; KT-ITZ, 97.9 ± 0.8%; and ND-ITZ 94.4 ± 1.8%) (*n* = 4).

### Stability tests

The stock solutions of ITZ, OH-ITZ, KT-ITZ, ND-ITZ, and ITZ*-d9* remained constant at 4 °C for up to 3 months. The contents of ITZ, OH-ITZ, KT-ITZ, and ND-ITZ in plasma were not degraded at room temperature (% of initial value, 92.6–101.3%) and 4 °C (99.2–102.9%) for up to 24 h. ITZ, OH-ITZ, KT-ITZ, and ND-ITZ remained unchanged at − 80 °C (90.3–101.5%) in plasma specimens for up to 1 month. ITZ, OH-ITZ, KT-ITZ, and ND-ITZ were stable at 4 °C (89.6–101.4%) in mobile phase for up to 12 h.

### Plasma concentrations of ITZ and its metabolites in patients

**Fig. 4:**
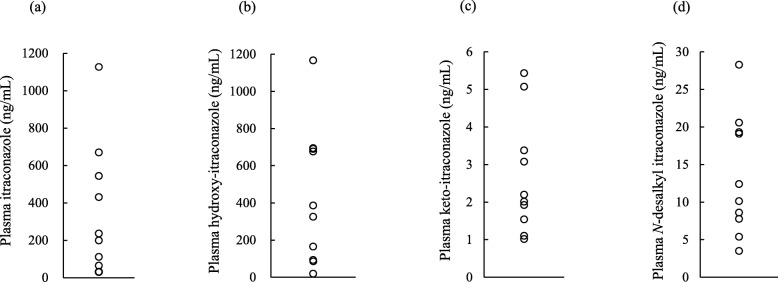
The distributions of plasma concentrations of (**a**) itraconazole, (**b**) hydroxy itraconazole, (**c**) keto itraconazole, and (**d**) *N*-desalkyl itraconazole in patients receiving 200 mg itraconazole daily

The plasma concentrations of ITZ, OH-ITZ, KT-ITZ, and ND-ITZ in patients treated with ITZ capsule formulation ranged from 32.5–1127.1, 19.0–1166.7, 1.1–5.4, and 3.5–28.3 ng/mL, respectively. The linearity ranges were wide enough to determine their plasma concentrations (Fig. 4). Table 3 shows the patient characteristics in this study population. The median age and serum creatinine values were 51 years and 0.79 mg/dl, respectively. No patient had significant liver dysfunction.

**Table 3:** Patient characteristics

Gender, male/female, n	7/3
Age, years	51 (44–62)
Body weight, kg	50.7 (47.6–56.6)
Body height, m	1.61 (1.57–1.69)
Serum total protein, g/dl	6.9 (5.0–7.3)
Serum albumin, g/dl	4.0 (3.7–4.3)
Serum creatinine, mg/dl	0.79 (0.72–0.91)
Blood urea nitrogen, mg/dl	16.4 (13.4–21.6)
Total bilirubin, mg/dl	0.6 (0.4–0.8)
Aspartate aminotransferase, IU/l	23 (19–25)
Alanine aminotransferase, IU/l	20 (15–46)
γ-Glutamyl transpeptidase, U/l	25 (19–41)

Data are expressed as median (interquartile range)

## Discussion

A simultaneous determination method for ITZ and its three major metabolites in human plasma using an isocratic LC-MS/MS system has been developed. We determined ITZ and its three metabolites including ND-ITZ in patient plasma using a simple isocratic LC with a common acetic acid and acetonitrile solution (pH 6.0) as the mobile phase coupled to MS/MS within 10 min using a 3-μm particle ODS column. Human plasma was pretreated by only protein precipitation with acetonitrile. This simpler preparation achieved higher recoveries. The plasma concentration ranges of ITZ and its metabolites in patients were within the range of each calibration curve. This method is simple for determining the plasma concentrations of ITZ and its three CYP3A4-mediated metabolites simultaneously in a clinical setting.

The present study detected symmetric ideal peaks. The injection volume of 2 μL also produced sharp peaks. We used 100 μL of plasma, and the LLOQs of ITZ, OH-ITZ, KT-ITZ, and ND-ITZ were 15, 15, 1, and 1 ng/mL, respectively. Some reports are available on the determination of ITZ and OH-ITZ [14, 16, 17, 19]. The LLOQs of ITZ, OH-ITZ, KT-ITZ, and ND-ITZ were 5, 5, 0.4, and 0.4 ng/mL in a previous report [15]. They used 150 μL of plasma, and the injection volume was 7 μL. Their calibration ranges of analytes (ITZ and OH-ITZ; 5–2500 ng/mL and KT-ITZ and ND-ITZ; 0.4–200 ng/mL) were slightly wider compared to those in the current study. Their LLOQs in this study were higher than previous reports. Their concentrations were above the LLOQs in 10 Japanese immunocompromised patients with a hematological disorder (Fig. 4), so the calibration ranges might be regarded as being wide enough to measure their concentrations in patient specimens. We verified the integrity of the dilution and found we can determine up to 6000 ng/mL ITZ and OH-ITZ and 400 ng/mL KT-ITZ and ND-ITZ. Our method may be more suitable for the determination of the plasma concentrations of ITZ and its three metabolites in both pharmacokinetic studies and clinical settings.

This study determined ND-ITZ in Japanese patients using isocratic elution on a conventional ODS column. This procedure is easier than gradient elution. The pH of the mobile phase was set at 6.0 because the pKa of ITZ was 3.7. If the pH of the mobile phase deviates from 3.7 by 2 or more, the peaks of ITZ and its metabolites will be sharp and distinct. The higher percentage of acetonitrile in this study made the run time shorter in spite of the 3-μm ODS column length of 7.5 cm. We used acetonitrile, which has not been classified as a carcinogenic reagent by the Globally Harmonized System of Classification and Labelling of Chemicals, in the preprocessing stage. There is another report on the determination of plasma ND-ITZ in healthy subjects. Liang et al. [15] pretreated human plasma with solid-supported liquid extraction using methyl tertiary butyl ether, which is classified as a carcinogen by the Globally Harmonized System of Classification and Labelling of Chemicals. Furthermore, the preparation is quite complicated. They determined ITZ, OH-ITZ, KT-ITZ, and ND-ITZ using gradient elution and an F5 column coupled to MS/MS in healthy subjects [15]. Gradient LC must use multiple pumps and mobile phases. The F5 column has a smaller particle in size resulting to higher pressure. Also, it needs chromatographic facilities and is less conventional. It is unclear whether their method can be clinically applicable because they did not measure patient specimens.

**Fig. 5:**
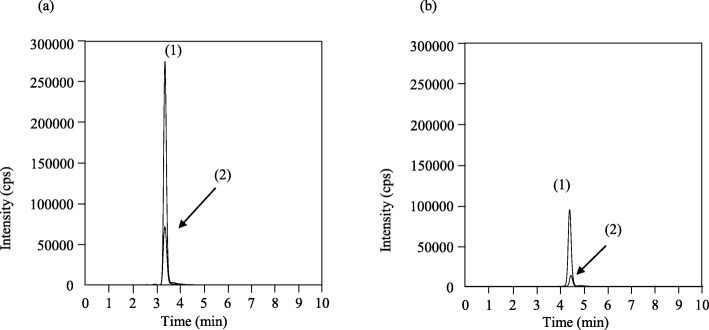
(**a**) The LC-MS/MS chromatograms of a plasma sample spiked with 300 ng/mL hydroxy itraconazole. The MRM transitions of (1) and (2) are 719.10/406.10 and 721.15/408.15, respectively. (**b**) The LC-MS/MS chromatograms of a plasma sample spiked with 20 ng/mL keto itraconazole. The MRM transitions of (1) and (2) are 721.15/408.15 and 719.10/406.10, respectively

There are several limitations in the present method. First, the blood concentration cannot be evaluated as the predose value, although the trough concentrations of ITZ and OH-ITZ have been reported as indicators of the preventive effect [4, 5, 20–22]. Patient plasma was obtained 12 h after administration, which was the same time as the daily clinical laboratory test. The concentration 12 h after administration may be slightly higher than the predose concentration. Second, we did not evaluate the present method with respect to its suitability for special populations. ITZ is eliminated mainly through hepatic metabolism. Metabolites including OH-ITZ are more hydrophilic than ITZ and may be renally excreted. The present method also needs to be verified in patients with severe renal impairment or hepatic dysfunction because the concentration ranges and the matrix effect are unknown. Third, unidentified peaks were obtained from the LC-MS/MS chromatograms (Fig. 5). From the aspect of MRM transitions, unknown peaks may be from an isotope of OH-ITZ or a KT-ITZ diastereomer. The determination of unidentified peaks will provide more concise information on ITZ metabolism. This method detects two isotopic peaks of OH-ITZ and KT-ITZ (Fig. 5). The isotopic peak is believed to be due to the chloride. It is important that ITZ contains two chlorides in its chemical structure to evaluate the isotopic peaks. The chloride are ^35^Cl:^37^Cl = 3:1. Peaks would be detected as M:M + 2:M + 4 = 9:6:1. The two chlorides seem to produce three isotopic peaks. The mass shift of product ions between OH-ITZ (*m/z* 408.5) and KT-ITZ (*m/z* 406.10) was almost 2.0 Da. This can be caused by an isotopic peak of OH-ITZ. In addition, a diastereomer of KT-ITZ was recently reported [23]. Unknown diastereomers may produce isotopic peaks.

## Conclusions

The present method with acceptable analytical performance was successfully applied for determination of the plasma concentrations of ITZ and its three CYP3A4-mediated metabolites in patients. The determination method appears to be more practical to those described in earlier reports in terms of conventionality and clinical adaptability.

## Data Availability

Data sharing not applicable to this article as no datasets were generated or analyzed during the current study.

## Abbreviations

CYP: cytochrome P450
IS: internal standard
ITZ: itraconazole
KT-ITZ: keto-itraconazole
LC-MS: liquid chromatography-mass spectrometry
LC-MS/MS: liquid chromatography-tandem mass spectrometry
LLOQ: lower limit of quantification
MRM: multiple reaction monitoring
MS/MS: tandem mass spectrometry
ND-ITZ: *N*-desalkyl itraconazole
ODS: octadecyl silane
OH-ITZ: hydroxy-itraconazole
QC: quality control
